# The increased anterior talofibular ligament–posterior talofibular ligament angle on MRI may help evaluate chronic ankle instability

**DOI:** 10.1007/s00276-023-03196-7

**Published:** 2023-07-10

**Authors:** Lei Zhang, Ting Lan, Junyao Chen, Zidong Wei, Houyin Shi, Guoyou Wang

**Affiliations:** 1 Department of Orthopedics, The Affiliated Traditional Chinese Medicine Hospital of Southwest Medical University, Luzhou, 646000 China; 2 Center for Orthopedic Diseases Research, The Affiliated Traditional Chinese Medicine Hospital of Southwest Medical University, Luzhou, 646000 China; 3 School of Clinical Medicine, Southwest Medical University, Luzhou, 646000 China

**Keywords:** Chronic ankle instability, Anterior talofibular ligament, Posterior talofibular ligament, Angle, MRI

## Abstract

**Purpose:**

This study intended to compare the difference between the anterior talofibular ligament (ATFL) and posterior talofibular ligament (PTFL) angle with chronic ankle instability (CAI) patients and healthy volunteers, and to confirm whether using the ATFL–PTFL angle could be a reliable assessment method for CAI, so as to improve the accuracy and specificity of clinical diagnosis.

**Methods:**

This retrospective study included 240 participants: 120 CAI patients and 120 healthy volunteers between 2015 and 2021. The ATFL–PTFL angle of the ankle region was gaged in the cross-sectional supine position on MRI between two groups. After participants undergoing a comprehensive MRI scanning, ATFL–PTFL angles were regarded as the main indicator of patients with the injured ATFLs and healthy volunteers to compare, and were measured by an experienced musculoskeletal radiologist. Moreover, other qualitative and quantitative indicators referring to anatomical and morphological characteristics of the AFTL were included in this study with MRI, such as the length, width, thickness, shape, continuity, and signal intensity of the ATFL, which can be used as secondary indicators.

**Results:**

In the CAI group, the ATFL–PTFL angle was 90.8° ± 5.7°, which was significantly different from the non-CAI group where the ATFL–PTFL angle for 80.0° ± 3.7° (*p* < 0.001). As for the ATFL-MRI characteristics, the length (*p* = 0.003), width (*p* < 0.001), and thickness (*p* < 0.001) in the CAI group were also significantly different from the non-CAI group. Over 90% of the cases, patients of the CAI group had injured ATFL with an irregular shape, non-continuous, and high or mixed signal intensity.

**Conclusion:**

Compared with healthy people, the ATFL–PTFL angle of most CAI patients is larger, which can be used as a secondary index to diagnose CAI. However, the MRI characteristic changes of ATFL may not relate to the increased ATFL–PTFL angle.

## Introduction

Ankle joint lateral collateral ligament (LCL) complex, including the ATFL, the PTFL, and the calcaneo-fibular ligament (CFL), has an extremely high incidence of injuries in daily living and sports activities, which accounts for 80–90% of ankle sprain [3, 5, 7, 8]. There are some typical symptoms after ankle sprain, such as pain, swelling, perception of instability, and so on [6, 7]. In general, after ankle sprain, about 10–40% of patients fail to conservative treatment and develop CAI, which can seriously damage their exercise function and reduce quality of life [2, 8, 14, 26, 29]. Among LCL complex, ATFL is the weakest ligament leading to frequent injury, yet PTFL and CFL are strong ligaments which are almost unaffected in ankle sprain [1, 7, 8, 12, 27, 29]. ATFL damage is an important mechanism of CAI [11].

Magnetic resonance imaging (MRI) can be used in the diagnosis and prognostic assessment of LCL complex injury [4, 10, 17], but it is not highly sensitive and accurate in diagnosing injured ATFLs. Therefore, some studies have pointed out that indirect signs can be used to increase the sensitivity and accuracy in diagnosing CAI, like the ATFL–PTFL angle [6, 15]. ATFL injury mostly causes the change of the structure of LCL complex, so injured ATFL could change the ATFL–PTFL angle. Accordingly, the ATFL–PTFL angle and the severity of ATFL damage would be estimated by MRI.

Inspired by Li et al. [23], we carried out further research based on their work. This study intends to compare the ATFL–PTFL angle of CAI patients and healthy volunteers and take ATFL-MRI characteristics as secondary indicators to explore the relationship between the two, so as to provide a new idea for the diagnosis of CAI, and improve its sensitivity and accuracy on the original basis. The hypothesis was that there are significant differences between the ATFL–PTFL angle of patients with CAI and normal people.

## Materials and methods

All study procedures were approved by the Ethical Committee of The Affiliated Traditional Chinese Medicine Hospital of Southwest Medical University (NO.KY2022041). CAI includes functional ankle instability, mechanical ankle instability, and recurrent ankle instability. Here patients with mechanical ankle laxity were included. One hundred and twenty CAI patients admitted to The Affiliated Traditional Chinese Medicine Hospital of Southwest Medical University from January 2015 to September 2021 would be included as the experimental group. An experienced orthopedist conducted history screening for ankle sprains, physical examination, varus pressure test, or front drawer test in the experimental group, and all the results suggested that the patients had suffered from CAI based on clinical symptoms, stress X-rays and MRI. The inclusion criteria were as follows: (1) Over the past year, there have been more than one and non-simultaneous cases of unilateral ankle sprains, most recently four weeks ago. (2) Patients with subjective sensation of ankle instability, persistent fatigue, pain during exercise, loss of function, or relaxation of ankle joint during assisted examination or MRI signs of ligament damage. The imaging data of the subjects were analyzed by two other doctors.

The control group consisted of 120 participants who had undergone MRI for reasons unrelated to CAI had been included and excluded by the same orthopedist. And the exclusion criteria were as follows: (1) previous ankle sprains; (2) a history of ankle surgery or a history of ipsilateral lower limb surgery; (3) malformation related to the ankle joint, for example, high arch, flat foot, inverting tibial plateau or varus heel; (4) previous history of ankle fracture; (5) PTFL tearing patients; (6) ATFL tearing patients and the ligament absorbed absolutely; (7) age more than 80 years or less than 18 years.

The demographic data of the participators are presented in Table 1. There were no significant differences in age, height, body weight, and body mass index between the control group (group non-CAI) and injured group (group CAI).

**Table 1:** Participant Demographic Data

Group	Subjects, *n*	Sex, *n* Male/Female	Age, years Range	Height, cm Range	Weight, kg Range	BMI Range
CAI	120	64/56	40.1 ± 17.4 (18–73)	163.9 ± 8.9 (142–188)	60.9 ± 10.3 (35–102)	22.6 ± 3.2 (15.8–32.6)
Non-CAI	120	67/53	41.7 ± 15.8 (18–80)	165.2 ± 8.3 (148–190)	62.3 ± 11.0 (42–110)	22.8 ± 3.2 (16.4–35.6)

### MRI scans and images analysis

All MRI examinations were performed using a three-dimensional MRI superconducting scanner (MAGNETOM Skyra, A Tim system, Siemens, Germany) with special coil adopted in a standardized fashion. In our radiology department, the standard protocol for ankle MRI is in a neutral position without any fixation applied in the supine position. Scanning sequence and parameters: axial T2WTSE sequence (TR 2840 ms, TE 66 ms, slick thickness 4 mm, FOV 230 mm), coronal PD FSE TS sequence (TR 2640 ms, TE 36 ms, slick thickness 3 mm, FOV 200 mm).

**Fig. 1:**
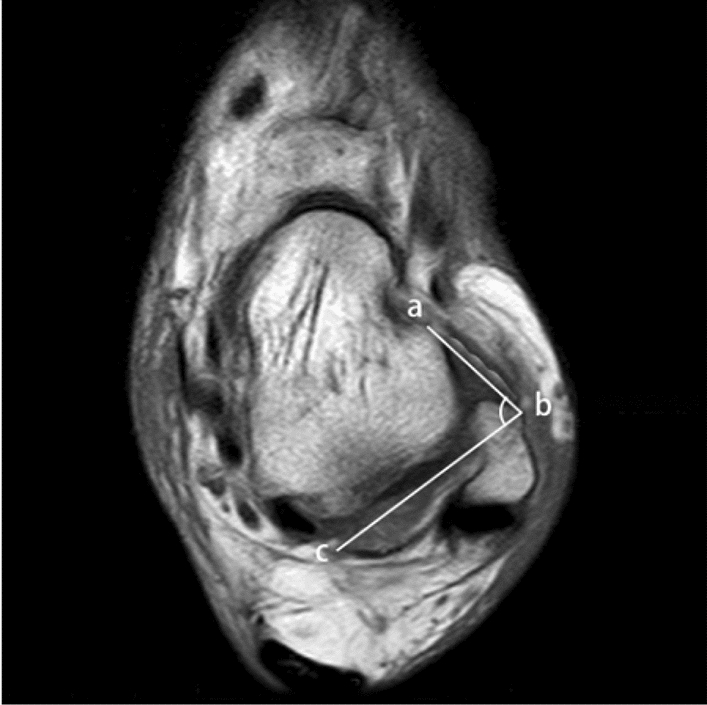
Line ab was drawn parallel to the anterior talofibular ligament (ATFL); line bc was drawn parallel to the posterior talofibular ligament (PTFL). The angle between line ab and line bc is the ATFL–PTFL angle

Previous researches indicated that the optimal position for MRI scanning is the supine position because of a long examination. In addition, the ATFL–PTFL angle of the ankle region can be displayed more clearly in the transverse axial plane [4, 16]. In all participators, the ATFL–PTFL angles were gaged in the following ways (Fig. 1). When the ATFL and PTFL appeared at the same time on the axial image, this plane was ascertained. The plane was the transverse section, and the shape of the fibula is crescent-shaped [24]. Two straight lines were drawn on the plane, one parallel to the ATFL and the other parallel to the PTFL. Be easy to see, the angle between the two lines was defined as the ATFL–PTFL angle, which would be gaged [12, 16].

**Fig. 2:**
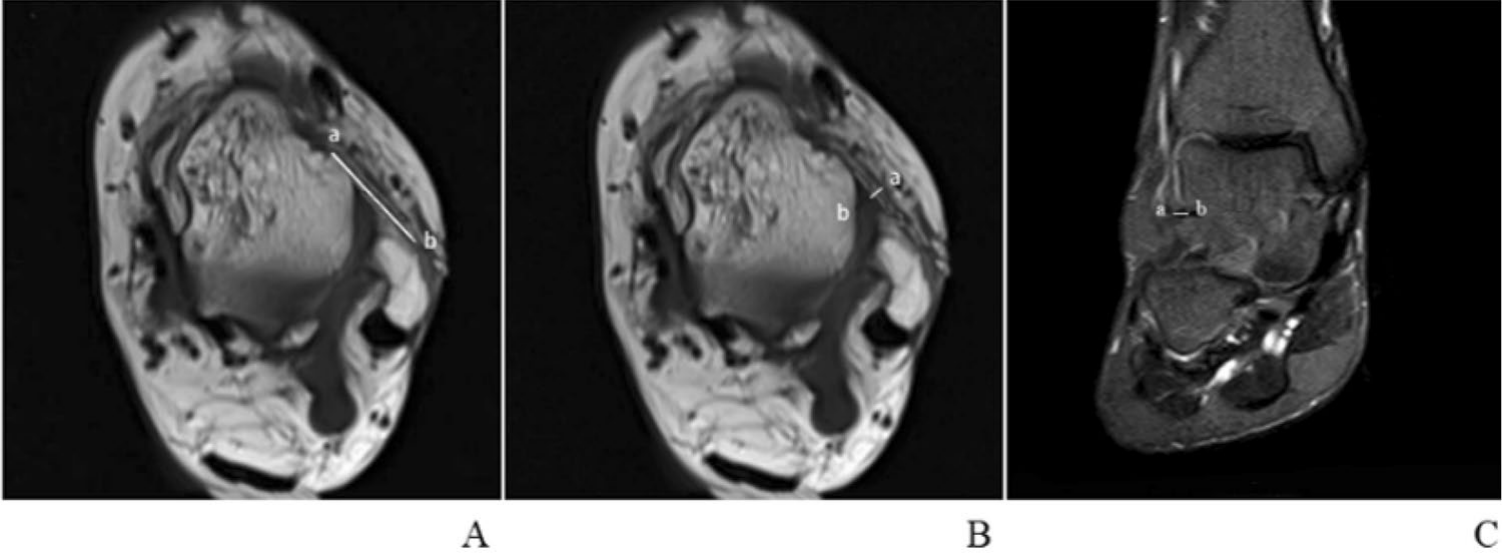
**A** In the cross section, a and b are the two stops of ATFL respectively, and the distance between the two points is the ATFL's length; **B** In the cross section, the first and last occurrence of the ATFL is its lower edge and upper edge, respectively, and the distance between the two, i.e. line ab, is the ATFL's width; **C** Measure the distance between talus and fibula in the coronal section, that is, line ab is the ATFL's thickness

In the control group, the length of the ATFL was also gaged in the transverse section, and the ATFL’s width was gaged at the midpoint of the total ligament length (Fig. 2 a, b). What’s more, the shape of the ATFL, the continuity, and signal intensity of the ATFL were also evaluated in this plane. But the thickness of the ATFL was gaged in the coronal section (Fig. 2c). In the CAI group, the mentioned above anatomical and morphological changes of the ATFL were gaged by the same way [16, 27]. The criteria of the injury ATFL for MRI assessments were as follows: (1) wavy or irregular appearance, (2) the presence of discontinuity, (3) enhanced signal intensity on T2-weighted images [4, 10, 19]. In the cross section, the location of the fibula was first determined. Then, at the level of column fibula, we could see a uniform continuous signal between tibia and fibula, namely ATFL. The distance between the two stops was the length of ATFL, and its width was gaged throughout the middle of the entire ligament. In the MRI cross section, when the ATFL first appears, this position is seen as its lower edge, and then as its upper edge by the last appearance, and we think of the distance between this as its width [9]. The injured ATFL had distorted morphology, interrupted continuity, irregular ligament morphology (adhesion or thinning), and uneven signal (high signal or mixed signal intensity). Tarsal sinus could be observed on the coronal plane of T2 image, where ATFL would appear as a small black dot connecting talus and fibula. Measure the distance between talus and fibula, that was, the thickness of ATFL.

Angle measurements, anatomical assessments, and morphological evaluations would put to use a picture archiving and PACS communication system. All measurements were accurate to one or two decimal places after the decimal point (angle with one decimal place, others with two decimal places). One independent observer was blinded to grouping, and gaged all the values of the MRI scan. Each survey was repeated at least three times to calculate the mean and the intra-observer analysis. In order to eliminate subjective consistency of data due to recall, the measurement interval was at least two weeks.

### Statistical analysis

All statistical analyses were performed using SPSS 26.0 (IBM Corporation, Armonk, New York, USA). All measurements were expressed as the mean and the standard deviation. Intra-observer measurement reliabilities were analyzed with the intra-class correlation coefficient (ICC). Receiver operating characteristic curves (ROC) were used to analyze the ATFL–PTFL angle of CAI patients and control group. And the sensitivity and specificity for evaluating were calculated. Since the data of the ATFL–PTFL angles were normally distributed, at the same time there was homogeneity of variance, the ATFL–PTFL angles between CAI patients and non-CAI individuals were compared by the independent t-test. In CAI patients, the relationship between the ATFL–PTFL angle and other MRI data was analyzed by Person correlation coefficient. P < 0.05 was deemed statistically significant. Post hoc analysis was used to evaluate the statistical power. The sample size was 120, the α level is 0.05, and the evaluation power is 1.0.

## Results

**Fig. 3:**
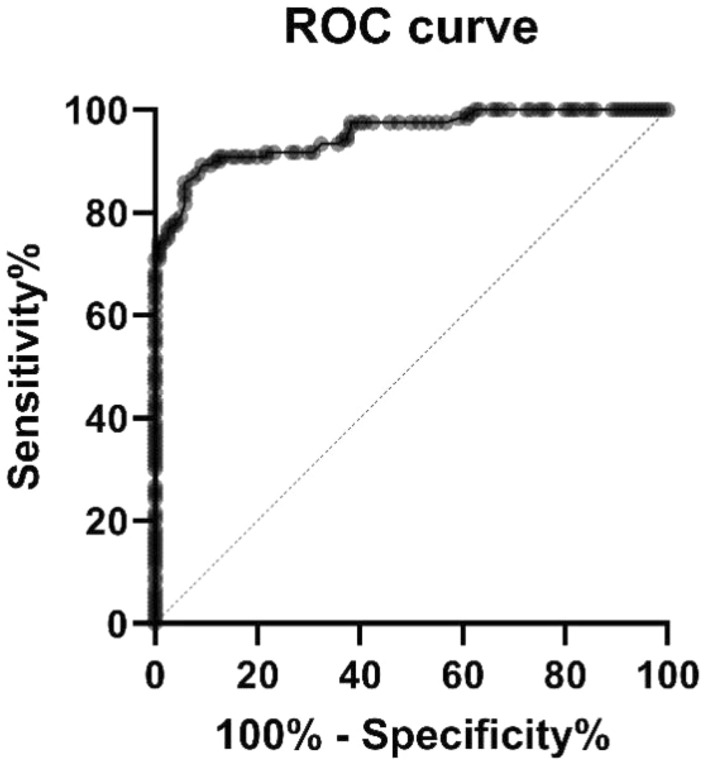
Examination of the ATFL–PTFL angle accuracy with Roc Curve test, with 95% confidence interval

The mean ATFL–PTFL angle in the group CAI was 90.8° ± 5.7°. All angles ranged from 79.3° to 103.9°, the range was 24.6°. The mean ATFL-PTFL angle in group non-CAI was 80.0° ± 3.7°. All angles ranged from 68.0° to 87.6°, the range was 19.4°. The ATFL–PTFL angle varied between two groups (*p* < 0.01), and the mean angle of the group CAI was significantly greater than that of the group non-CAI. In the group CAI, as for the ATFL-MRI characteristics, the length, the width, and the thickness were also significantly different from the group non-CAI. They were longer, narrower, and thinner in the group CAI. Over 90% of the patients, patients of group CAI had injured ATFL with irregular shape, non-continuous, and high or mixed signal intensity (Table 2). In CAI patients, there was no significant correlation among the length (*r* = 0.236, *p* < 0.001), width (*r* = -0.474, *p* < 0.001), thickness (*r* =  − 0.383, *p* < 0.001), and the ATFL–PTFL angle. The intra-observer and inter-observer reliabilities for all measurements were good (*p* < 0.001) (Table 3). The area under the ROC was 0.952 (*p* < 0.001). The best ROC curve truncation point for ATFL injury diagnosis is ATFL–PTFL angle 85.05°, sensitivity is 0.858, and specificity is 0.942 (Fig. 3).

**Table 2:** ATFL–PTFL Angle and Other Indicators

Group	ATFL–PTFL angle Range	Length, cm Range	Width, cm Range	Thickness, cm Range	Shape, *n* Irregular /standard	Continuity, *n* Inconsecutive /consecutive	Signal intensity, *n* Hich or mixed /middle
CAI	90.8° ± 5.7° (79.3°-103.9°)	2.19 ± 0.21 (1.41–2.92)	0.25 ± 0.04 (0.15–0.37)	0.22 ± 0.05 (0.08–0.40)	114/6	120/0	108/12
Non-CAI	80.0° ± 3.7° (68.0°–87.6°)	2.12 ± 0.17 (1.72–2.44)	0.35 ± 0.08 (0.17–0.55)	0.27 ± 0.06 (0.18–0.45)	16/104	4/116	4/116
*P* value	0.000	0.003	0.000	0.000

**Table 3:** Reliability of MRI measurement evaluated with intra-class correlation coefficient

		ATFL–PTFL angle	Length	Width	Thickness
Interobserver agreement		0.928 (0.908, 0.944)	0.957 (0.945, 0.966)	0.941 (0.924, 0.954)	0.924 (0.904, 0.941)
Intra-observer reproducibility	Researcher 1	0.885 (0.854, 0.910)	0.850 (0.810, 0.881)	0.777 (0.721, 0.822)	0.781 (0.726, 0.826)
	Researcher 2	0.931 (0.912, 0.946)	0.937 (0.920, 0.951)	0.818 (0.771, 0.856)	0.961 (0.950, 0.970)

## Discussion

The main finding of this study was that CAI patients have the larger ATFL–PTFL angle than normal participants. And the length, the width, and the thickness of ATFL were longer, narrower, and thinner in the CAI patients.

In general, after ankle sprain, about 10–40% of patients fail in conservative treatment and develop into CAI, which can seriously damage their exercise function and reduce quality of life [2, 8, 14, 23, 26, 29]. Therefore, accurate assessment of the ATFL is vital when orthopedists choose treatment prescriptions. The authority of MRI in assessing damaged ligaments has been confirmed in numerous studies [6, 15, 17]. However, Li et al. have questioned the accuracy and the sensitivity of MRI in diagnosing ATFL damage [23]. In the meta-analysis, Cao et al. proposed that the combined accuracy and sensitivity of MRI in diagnosing ATFL damage are 0.83 and 0.79, respectively [6]. Furthermore, both the accuracy and sensitivity of MRI were decreased in the case of patients with partial ligament tears. On the one hand, soft tissue swelling, external malleolus space bleeding, and joint space bleeding make MRI diagnosis more difficult. On the other hand, to some extent, the assessment of ligament damage is influenced by the experience of radiologists. To sum up, it is necessary for us to put forward an easy and novel approach to improve the accuracy and sensitivity of diagnosing LCL complex damage. It has been pointed out that ATFL-PTF angle can be used as a new indirect MRI feature for diagnosing ATFL damage [23]. The measurement and detection of this angle are highly reproducible and easy to operate on MRI images, which indicates great value in clinical practice. The reasons why the ATFL–PTFL angle increased in CAI patients are unknown. One possible reason may be that ATFL gets distorted and loses after damage, but PTFL usually keeps in a good condition, and thus lead to a larger angle between them. Another possible reason is the location change of the talus. In a systematic review, it was found that CAI ankles had significant forward movement and internal rotation. But there is no consensus on fibula alignment or foot posture. As a result of this, ATFL and PTFL stop relative position change, causing an increase in the angle between ATFL and PTFL [21]. Although this retrospective study revealed significant differences between group CAI and group non-CAI in the ATFL–PTFL angle, an increase in ATFL–PTFL angle did not always appear in CAI patients. The ATFL–PTFL angle was less than 80.0° in 21 patients in the CAI group. In addition, there was extensive variability in ATFL–PTFL angles (ranges of 24.6° and 19.4° in the CAI group and the control group, respectively). Consequently, the ATFL–PTFL angle cannot be used as a gold standard for assessing ATFL damage. The relationship between CAI and these changes needs further investigation.

Based on studies by Li et al. [23], further studies with more innovation and clinical relevance were carried out. We did not use the ATFL–PTFL angle as a direct standard for CAI diagnosis, but as a major indicator. Moreover, ATFL–PTFL angle combined with ATFL-MRI characteristics that regarded as an auxiliary indicator, connected the status of ATFL with CAI, which was more accurate and specific for CAI diagnosis and had more clinical significance in clinical work than these of Li et al. [23]. Although in our study, the ATFL–PTFL angle and ATFL-MRI characteristics are not highly relevant. In Li et al. study, the ATFL–PTFL angle differs from our results by nearly 10 degrees. For this reason, we speculate that first Li et al. was included in all of the patients with MAI, and then the sex ratio was different, with more men in it. This may be responsible for different perspectives. Clinicians can mainly use the ATFL–PTFL angle to diagnose CAI. In addition, the ATFL-MRI characteristics can also be used to reduce the CAI misdiagnosis rate and missed diagnosis rate. It is conducive to providing patients with personalized and effective clinical treatment plans. The precision medical goal will go further, and this is a win–win situation for clinicians and patients.

Various techniques, including ultrasonography and histological examinations, had been investigated to study the differences in the fibular attachment structure of the anterior talofibular ligament (ATFL). The study conducted by Kim et al. [20]. focuses on the morphological changes in the talofibular ligaments during fetal development and growth. This research provides valuable insights into the structural variations and developmental aspects of the ligaments, which can have implications for understanding ligament laxity and its potential impact on ankle sprains. The findings may contribute to the interpretation of the study results, considering the dynamic nature of ligament development and potential differences in ligament characteristics among different age groups. Another study explored the relationship between the inferior fascicle of the ATFL and the articular capsule within the lateral ankle ligament complex [19]. These studies provide valuable insights into the anatomical variations and developmental changes in talofibular ligaments, which are essential in addressing the presence of patients with ligament laxity and understanding its impact on the study results.

For orthopedic medicine world, in terms of imaging examination, we recommend using the ATFL–PTFL angle as the primary indicator for the initial diagnosis of CAI. When the diagnosis is unknown or a large rate of angular change is observed in individual patients, the ATFL-MRI characteristics can be taken as a secondary index, which partly improves the sensitivity and specificity of confirmed CAI. It is a rapid and accurate diagnosis for orthopedic surgeons and a non-invasive examination for patients. In addition, orthopedic surgeons can choose the treatment plan through the size of the ATFL–PTFL angle, perform conservative treatment or surgical treatment, but this cut-off value needs further research.

## Limitations

There are some limitations to this study. MRI scans were obtained in a supine position, a non-weight-bearing position. Since the ATFL–PTFL angle may vary in the weight-bearing and non-loading position, the ATFL–PTFL angle can be changed. Nevertheless, all patients in this study underwent scanning in the same posture. Accordingly, the measurement results between the two groups were considered comparable. Further study in weight-bearing positions of ankles will be required in future. Furthermore, the CAI is defined far beyond ligament laxity, though pathologic laxity of the ankle was the initial cause of the functional instability of the foot [16, 22]. After adding the detailed inclusion criteria, our study mainly based on CAI patients, which also included some patients with ligament laxity after ankle sprains. Thus, a clearer definition of CAI and a rigorous screening are needed.

## Conclusion

ATFL–PTFL angle provides a novel and indirect MRI signature for CAI diagnosis. Compared with healthy individuals, most CAI patients have a larger ATFL–PTFL angle, which can assist in the diagnosis of CAI. But the MRI characteristic changes of ATFL may not relate to the increased ATFL–PTFL angle.

## Data Availability

The data that support the findings of this study are available on request from the corresponding author.

## References

[1] An L, Yan Y (2021). MRI-based diagnosis of anterior talofibular ligament injury. J Coll Physicians Surg Pak.

[2] Ahn J, Choi JG, Jeong BO (2021). The signal intensity of preoperative magnetic resonance imaging has predictive value for determining the arthroscopic reparability of the anterior talofibular ligament. Knee Surg Sports Traumatol Arthrosc.

[3] Altmeppen JN, Colcuc C, Balser C (2022). A 10-year follow-up of ankle syndesmotic injuries: prospective comparison of knotless suture-button fixation and syndesmotic screw fixation. J Clin Med.

[4] Alvarez C, Hattori S, Kato Y (2020). Dynamic high-resolution ultrasound in the diagnosis of calcaneofibular ligament injury in chronic lateral ankle injury: a comparison with three-dimensional magnetic resonance imaging. J Med Ultrason.

[5] Barini M, Zagaria D, Licandro D (2021). Magnetic Resonance accuracy in the diagnosis of anterior talo-fibular ligament acute injury: a systematic review and meta-analysis. Diagnostics (Basel).

[6] Cao S, Wang C, Ma X (2018). Imaging diagnosis for chronic lateral ankle ligament injury: a systemic review with meta-analysis. J Orthop Surg Res.

[7] Cao S, Wang C, Ma X (2019). Reliability and validity of different ankle MRI scanning planes for the anterior talofibular ligament injury diagnosis: a cadaveric study. J Orthop Surg Res.

[8] Casado-Hernández I, Becerro-de-Bengoa-Vallejo R, Losa-Iglesias ME (2021). Association between anterior talofibular ligament injury and ankle tendon, ligament, and joint conditions revealed by magnetic resonance imaging. Quant Imaging Med Surg.

[9] Choi SM, Cho BK, Kim SH (2022). The Influence of suture-tape augmentation on biological healing of the anterior talofibular ligament in chronic ankle instability: a quantitative analysis using MRI. J Foot Ankle Surg.

[10] Cho JH, Lee DH, Song HK (2016). Value of stress ultrasound for the diagnosis of chronic ankle instability compared to manual anterior drawer test, stress radiography, magnetic resonance imaging, and arthroscopy. Knee Surg Sports Traumatol Arthrosc.

[11] Dalmau-Pastor M, El-Daou H, Stephen JM, Vega J, Malagelada F, Calder J (2023) Clinical relevance and function of anterior talofibular ligament superior and inferior fascicles: a robotic study. Am J Sports Med. 3635465231172196. 10.1177/03635465231172196 37232327

[12] Dalmau-Pastor M, Malagelada F, Calder J (2020). The lateral ankle ligaments are interconnected: the medial connecting fibres between the anterior talofibular, calcaneofibular and posterior talofibular ligaments. Knee Surg Sports Traumatol Arthrosc.

[13] Drakonaki EE, Gataa KG, Solidakis N (2021). Anatomical variations and interconnections of the superior peroneal retinaculum to adjacent lateral ankle structures: a preliminary imaging anatomy study. J Ultrason.

[14] Ma D, Hansen O, Kukadia S (2022). Ankle Instability. Foot Ankle Clin.

[15] Gribble PA (2019). Evaluating and differentiating ankle instability. J Athl Train.

[16] Hertel J, Corbett RO (2019). An updated model of chronic ankle instability. J Athl Train.

[17] Jolman S, Robbins J, Lewis L (2017). Comparison of magnetic resonance imaging and stress radiographs in the evaluation of chronic lateral ankle instability. Foot Ankle Int.

[18] Jung HG, Kim NR, Kim TH (2017). Magnetic resonance imaging and stress radiography in chronic lateral ankle instability. Foot Ankle Int.

[19] Kakegawa A, Fukushima N, Sumitomo N, Nagira A, Ichinose Y, Moriizumi T (2022). Relationship between inferior fascicle of anterior talofibular ligament and articular capsule in lateral ankle ligament complex. Surg Radiol Anat.

[20] Kim JH, Jin ZW, Hayashi S, Murakami G, Rodríguez-Vázquez JF, Abe H (2022). Major change in morphology of the talofibular ligaments during fetal development and growth. Surg Radiol Anat.

[21] Kobayashi T, Koshino Y, Miki T (2021). Abnormalities of foot and ankle alignment in individuals with chronic ankle instability: a systematic review. BMC Musculoskelet Disord.

[22] Lalevée M, Anderson DD, Wilken JM (2023). Current challenges in chronic ankle instability: review and perspective. Foot Ankle Clin.

[23] Li HY, Li WL, Chen SY (2020). Increased ATFL-PTFL angle could be an indirect MRI sign in diagnosis of chronic ATFL injury. Knee Surg Sports Traumatol Arthrosc.

[24] Nazarenko A, Beltran LS, Bencardino JT (2013). Imaging evaluation of traumatic ligamentous injuries of the ankle and foot. Radiol Clin North Am.

[25] Szaro P, Ghali GK, Polaczek M (2020). The double fascicular variations of the anterior talofibular ligament and the calcaneofibular ligament correlate with interconnections between lateral ankle structures revealed on magnetic resonance imaging. Sci Rep.

[26] Szaro P, Ghali GK, Solidakis N (2021). Morphometric relationships between dimensions the anterior talofibular ligament and calcaneofibular ligament in routine magnetic resonance imaging. J Exp Orthop.

[27] Teramoto A, Akatsuka Y, Takashima H (2020). 3D MRI evaluation of morphological characteristics of lateral ankle ligaments in injured patients and uninjured controls. J Orthop Sci.

[28] Wenning M, Gehring D, Lange T (2021). Clinical evaluation of manual stress testing, stress ultrasound and 3D stress MRI in chronic mechanical ankle instability. BMC Musculoskelet Disord.

[29] Xu Y, He L, Han Y (2021). Evaluation of 3-Dimensional Magnetic Resonance Imaging (3D MRI) in Diagnosing Anterior Talofibular Ligament Injury. Med Sci Monit.

[30] Yan W, Meng X, Sun J (2021). Intelligent localization and quantitative evaluation of anterior talofibular ligament injury using magnetic resonance imaging of ankle. BMC Med Imaging.

